# Why Most Biomedical Findings Echoed by Newspapers Turn Out to be False: The Case of Attention Deficit Hyperactivity Disorder

**DOI:** 10.1371/journal.pone.0044275

**Published:** 2012-09-12

**Authors:** François Gonon, Jan-Pieter Konsman, David Cohen, Thomas Boraud

**Affiliations:** 1 Institute of Neurodegenerative Diseases, University of Bordeaux, Bordeaux, France; 2 Centre Nationale de la Recherche Scientifique UMR 5293, Bordeaux, France; 3 Centre de Résonance Magnétique des Systèmes Biologiques, University of Bordeaux, Bordeaux, France; 4 Centre Nationale de la Recherche Scientifique UMR 5536, Bordeaux, France; 5 School of Social Work, Florida International University, Miami, Florida, United States of America; University Paris Descartes, France

## Abstract

**Context:**

Because positive biomedical observations are more often published than those reporting no effect, initial observations are often refuted or attenuated by subsequent studies.

**Objective:**

To determine whether newspapers preferentially report on initial findings and whether they also report on subsequent studies.

**Methods:**

We focused on attention deficit hyperactivity disorder (ADHD). Using *Factiva* and *PubMed* databases, we identified 47 scientific publications on ADHD published in the 1990s and soon echoed by 347 newspapers articles. We selected the ten most echoed publications and collected all their relevant subsequent studies until 2011. We checked whether findings reported in each “top 10” publication were consistent with previous and subsequent observations. We also compared the newspaper coverage of the “top 10” publications to that of their related scientific studies.

**Results:**

Seven of the “top 10” publications were initial studies and the conclusions in six of them were either refuted or strongly attenuated subsequently. The seventh was not confirmed or refuted, but its main conclusion appears unlikely. Among the three “top 10” that were not initial studies, two were confirmed subsequently and the third was attenuated. The newspaper coverage of the “top 10” publications (223 articles) was much larger than that of the 67 related studies (57 articles). Moreover, only one of the latter newspaper articles reported that the corresponding “top 10” finding had been attenuated. The average impact factor of the scientific journals publishing studies echoed by newspapers (17.1 n = 56) was higher (p<0.0001) than that corresponding to related publications that were not echoed (6.4 n = 56).

**Conclusion:**

Because newspapers preferentially echo initial ADHD findings appearing in prominent journals, they report on uncertain findings that are often refuted or attenuated by subsequent studies. If this media reporting bias generalizes to health sciences, it represents a major cause of distortion in health science communication.

## Introduction

Because the mass media are a key source of health science information for the lay public and for many professionals, the accuracy of media reporting is a matter of concern. Numerous studies have investigated how the media report on single biomedical studies. Depending on medias and topics, the reporting accuracy ranges from poor to more accurate than expected [1]–[3]. However, “assessing accuracy in the reporting of a single study does not address whether the coverage contextualizes, where the study fits within an emerging body of knowledge” [4]. Biomedical findings slowly mature from initial uncertain observations to facts validated by subsequent independent studies [5]. Therefore, high quality media reporting of biomedical issues should consider a body of scientific studies over time, rather than merely initial publications [4]. This is all the more desirable since initial biomedical findings are often contradicted or attenuated by subsequent studies [6]–[8]. This devaluation trend is not surprising, from a scientific point of view, given that positive results are more often published than negative ones [6], [9].

We hypothesize here that the devaluation trend of initial findings is largely ignored by the media. Indeed, because of their novelty, initial observations tend to be published in prestigious scientific journals [6], [8] and, although data are still lacking, it is likely that most subsequent studies are published in less prestigious ones. If media preferentially report on findings published in prestigious journals, they may fail to reflect the scientific progress from initial observations to high-quality evidence based on sets of consistent scientific studies.

We focused on attention deficit hyperactivity disorder (ADHD), which is considered to be the most common neurodevelopmental disorder diagnosed in children, with a prevalence around 10% among children aged 4 to 17 years in the United States [10]. It is characterized by behavioral symptoms, mainly attention deficit and impulsivity with or without hyperactivity. The ADHD diagnosis rests only on these symptoms because no biological markers (e.g. genetic tests, brain imaging) have been validated [11]. Short-term studies have demonstrated that psychostimulant medications significantly reduce ADHD symptoms [11], [12]. However, according to recent reports, psychostimulant treatment of ADHD-diagnosed children does not decrease long-term risks of later antisocial behavior, substance use disorders and significant academic underachievement [13]–[15]. Debates about the diagnosis and treatment of ADHD persist in Europe and the USA [16].

To test our hypothesis, we selected the 10 scientific publications related to ADHD that were most frequently echoed by English-language newspapers during the 1990s. For each of these “top 10” studies we collected all subsequent scientific articles on the same specific topic as well as previous ones published in that decade. For every publication, we noted the impact factor of the journal that published it, the ranking of the university where the research was performed, and the number of newspaper articles that reported on it. We checked whether findings in each “top 10” publication were consistent with subsequent observations on the same specific topic until 2011. We also compared the newspaper coverage of the “top 10” publications to that of their related scientific studies.

## Methods

### Selection of “top 10” scientific publications

**Figure 1:**
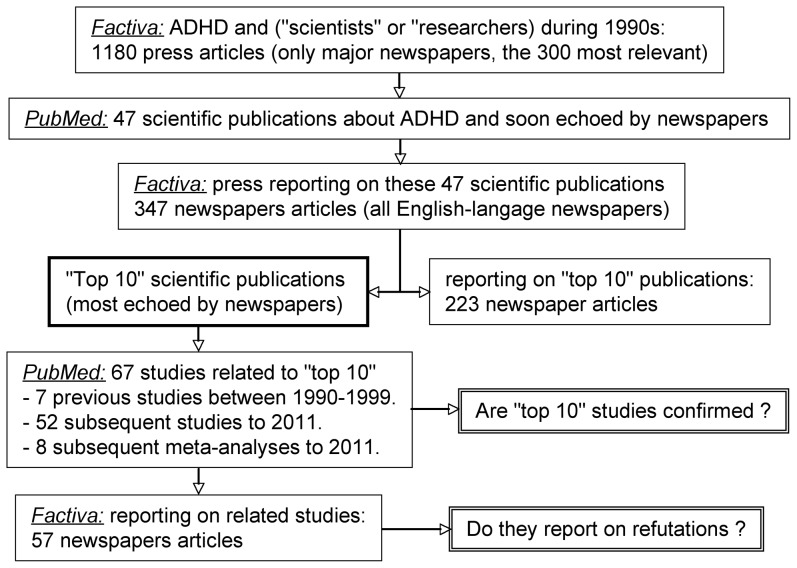
Design of the study. The databases used in each step are indicated in *italic*. Details are given in the methods.

The design of our study is illustrated in Fig. 1. We used the *Dow Jones Factiva* database to locate scientific publications on ADHD reported in English-language newspapers. This systematic search was performed in two steps. First, we conducted a Boolean search of the *Factiva* database using the keywords (hyperactivity OR ADHD OR attention deficit) AND (researcher* OR scientist*) applied from January 1, 1990 to December 31, 1999 within the restricted sources “Major News and Business Publications”. This produced 1180 articles. We sorted these by relevance using the *Factiva* tool and read the 300 most relevant ones to identify the scientific publications they echoed. These scientific publications were unequivocally identified in *PubMed* when their reference or author's name was given by the corresponding newspaper. In a few cases where such details were lacking, we identified the corresponding scientific publication by other details (university where the study was conducted, date of publication, numerical data). This first step retrieved 56 scientific publications. Among them one meta-analysis, two review articles, one opinion article and five articles only mentioning ADHD incidentally (e.g. a study focused on Tourette's syndrome) were not considered further. The remaining 47 publications reported on primary observations. They are listed as Table S1 with their respective number of associated newspaper articles.

In the second step we looked for all newspaper articles reporting on these 47 scientific publications. The following keyword Boolean search (hyperactivity OR ADHD OR attention deficit) was applied to the *Factiva* database without restriction regarding the source (“All sources”) within a 10-day time range starting one day before the publication date of every scientific publication published in weekly journals. For scientific publications published in monthly journals our procedure included a preliminary step. First, the time range was extended to two months and we looked for the earliest occurrence of a *Factiva* article published by any press agency (e.g. *Reuters News*, *Associated Press*). The date of this early article was, then, taken as the starting date of a 10-day time range, within which we performed the same systematic search. We included newspaper articles from the printed general press, but excluded articles from press agencies or published by specialized weekly magazines (e.g. *Biotech Week*, *Pharma Business Week*). We counted the number of newspaper articles echoing on each of the 47 scientific publications and ranked each publication by this number.

### Previous and subsequent scientific articles

For each “top 10” publication, we systematically searched *PubMed* to identify any previous related scientific publications appearing between 1990 and 1999, and any subsequent publications appearing until December 2011. Publications were selected if they provided experimental observations on the specific topic investigated in the corresponding “top 10” publication. To verify that we did not miss relevant publications we also selected meta-analyses covering the same topic using the *PubMed* limit “*meta-analysis*”. The ten boolean searches and the meta-analyses they yielded are given in Table S2. For each identified publication we used the *Factiva* database to look for newspaper articles reporting on it according to the procedure described above. Searches concluding that a scientific publication was not echoed by newspapers were checked independently by two authors.

### Content analysis of “top 10” publications and of their related newspaper articles

In each “top 10” publication we identified the major finding emphasized by most corresponding newspaper articles. Then, we checked whether it agreed with findings published in related studies. Whenever possible, deciding whether “top 10” findings were confirmed, attenuated or refuted was based on systematic review articles and meta-analyses published since 2008 and listed in Table S2. When recent meta-analyses were not found, either we considered the most recent study published by the same group when it refuted the initial claim (Table S2), or we checked the literature cited by articles published since 2008 on the same topic (see our detailed account as Text S1). Our judgment was performed in two steps by two authors familiar with the ADHD literature. First, one author wrote a preliminary evaluation of each “top 10” article on the basis of the corresponding related studies. Second, another author carefully checked this evaluation. Then, both authors resolved their few disagreements by discussion and built a detailed account of their judgment (Text S1). A brief account is given below.

For the nine “top 10” publications questioned or refuted by subsequent studies, we found 36 newspaper articles reporting on these subsequent studies, including meta-analyses. We checked whether any newspaper article mentioned that the scientific publication they echoed actually questioned or refuted the corresponding previous “top 10” publication. Because this analysis was partly subjective, it was independently performed by two coders. However, we observed no disagreement between them.

### Journal impact factor and university ranking

We characterized each scientific publication by the impact factor of the journal that published it and by the ranking of the university in which the study was performed. For publications with authors from different universities, the university indicated in the address of the corresponding author was selected. The impact factor was given by *ISI Web of Knowledge* using the *Journal Citation Reports*. We selected the 2009 edition and the 5-year impact factor. To quantify the ranking of each university we used the 2010 edition of the freely available *Academic Ranking of World Universities* (*“Shanghai Ranking”* http://www.arwu.org) where the best-ranked is assigned a score of one. We selected the ranking in *Clinical Medicine and Pharmacy*. Because rankings in the ranges of 51–75 and 76–100 were not specified, we assigned the arbitrary score of 63 and 88, respectively. Any university not classified among the top 100 was given the arbitrary score of 120. The National Institutes of Health in Bethesda, Maryland, are not listed in the “Shanghai Ranking”. Because of their prominence, however, we assigned them a score of one.

## Results

### Identification and characteristics of “top 10” articles

From our initial search we retained 47 scientific publications reporting primary observations related to ADHD and echoed at least once by newspapers during the nine days following their publication date. On average each publication was echoed by 7.4 newspaper articles (range: 1 to 37), but this media coverage was unevenly distributed. Each “top 10” publication, listed in Table 1, received an average of 22.3 newspaper articles (range: 13 to 37) whereas the 37 others were echoed by 3.4 newspaper articles each on average (range: 1 to 10) (Table 1 and Table S1). Examples of newspaper titles are given in Table 1 and are highly representative of all newspaper titles dealing with each “top 10” publication (data not shown). Among these “top 10” publications, four reported on neurobiological [17]–[20] and four on behavioral observations in humans, [21]–[24], one on neurobiological and behavioral observations in mice [25] and one on epidemiological data [26]. All but two appeared in highly prestigious journals, with an average 2009 impact factor of 26.6 (range: 5.8 to 51.4) (Table 1).

**Table 1:** “Top 10” scientific studies published between 1990 and 1999 and most frequently echoed by newspapers.

Year	1^st^ author	Scientific Title. *Typical newspaper title*	Journal. *Newspaper*	Impact factor	Media coverage
1990	Zametkin	Cerebral glucose metabolism in adults with hyperactivity of childhood onset. *Hyperactivity linked to brain dysfunction.*	N Engl J Med. *Houston Chronicle*	51.4	18
1993	Hauser	Attention deficit-hyperactivity disorder in people with generalized resistance to thyroid hormone. *Hyperactivity linked to genetic defect.*	N Engl J Med. *San Francisco Chronicle*	51.4	26
1994	Wolraich	Effects of diets high in sucrose or aspartame on the behavior and cognitive performance of children. *Sweeteners-hyperactivity link is discounted.*	N Engl J Med. *The New York Times*	51.4	24
1996	Lahoste	Dopamine D4 receptor gene polymorphism is associated with attention deficit hyperactivity disorder. *Genetic flaw linked to hyperactivity.*	Mol Psychiatry. *Chicago Sun-Times*	13	19
1996	Safer	Increased methylphenidate usage for attention deficit disorder in the 1990s. *Study sees lower increase in Ritalin use.*	Pediatrics. *The Dallas Morning News*	5.8	23
1998	Vaidya	Selective effects of methylphenidate in attention deficit hyperactivity disorder: a fMRI study. *Scan can diagnose kids' ADD.*	PNAS. *Times Union*	10.3	29
1999	Biederman	Pharmacotherapy of attention-deficit/hyperactivity disorder reduces risk for substance use disorder. *Ritalin users may be less likely to abuse drugs.*	Pediatrics. *Denver Post*	5.8	18
1999	Dougherty	Dopamine transporter density in patients with attention deficit hyperactivity disorder. *Brain scans seen as test in attention disorder.*	Lancet. *The Boston Globe*	29.4	37
1999	Gainetdinov	Role of serotonin in the paradoxical calming effect of psychostimulants on hyperactivity. *Better attention deficit drugs possible.*	Science. *The Washington Post*	31.1	12
1999	MTA group	A 14-month randomized clinical trial of treatment strategies for ADHD. *Medicine best help for ADD.*	Arch Gen Psychiatry. *The Cincinnati Post*	16.4	17

### Scientific follow-up of “top 10” publications reporting on neurobiological observations

All four publications providing neurobiological data in humans were initial studies [17]–[20]. Indeed, according to the authors and in agreement with our own searches, the specific questions under investigation were not previously tackled. In 1990 Zametkin and coworkers used positron-emission tomography (PET) to measure cerebral glucose metabolism in 25 adults with ADHD and in 50 normal adults while they performed an auditory task [17]. They reported that mean global cerebral glucose metabolism was 8.1% lower in the ADHD adults than in the normal controls. However, 4 subsequent studies from the same group fully disconfirmed the initial finding [27]–[30].

**Figure 2:**
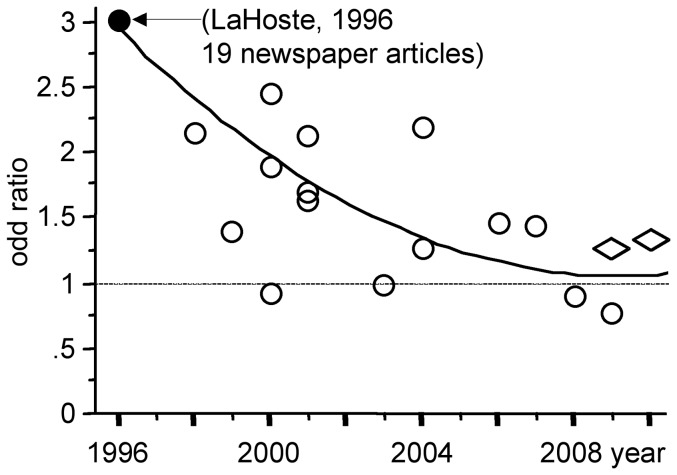
Association between ADHD and the D4 dopaminergic receptor gene in initial and subsequent studies. The strength of the association (i.e. the odd ratio, the ratio of prevalence of the 7R allele of this DRD4 gene in ADHD children versus control children) in each study is given in the ordinate. In 1996 LaHoste and coworkers published the first study associating ADHD with the DRD4 gene [18]. This initial study reported an odd ratio of 3.01. Subsequent case-control studies reported smaller odd ratio (data from table 1A in [47]). According to two recent meta-analyses [46], [47], ADHD is still considered to be significantly associated with the DRD4 gene although this association confers a much smaller risk than initially thought (odd ratio indicated by a diamond). Filled circles indicate scientific studies echoed by newspapers (number of media articles indicated in parentheses). Empty circles and diamonds indicate that the publication has not been echoed by newspapers.

LaHoste and coworkers showed in 1996 that the “7R” allele of the gene coding for the D4 dopamine receptor was present in 49% of ADHD children and in only 21% of the healthy control children [18]. Fifteen subsequent investigations of this case-control association, however, did not confirm this large difference (Fig. 2 and Table 2) [31]–[45]. Indeed, meta-analyses have repeatedly concluded that the 7R allele confers a statistically significant, but small, risk: it is present in 23% of ADHD children and in 17% of controls [46], [47].

**Table 2:** “Top 10” publications and their related scientific studies.

“Top 10		Related studies				Meta-analyses			Scientific follow-up
Year	1^st^ author	refuting or attenuating *Ref*	supporting *Ref*	IF, mean ±SD	Echoed (total) #	*Ref*	IF	Echoed #	
1990	Zametkin	[*27*] *, * [*28*] *, * [*29*] *, * [*30*]		7.7±5.9	0				Refuted
1993	Hauser	[*65*] *, * [*66*] *, * [*67*] *, * [*68*] *, * [*69*] *, * [*70*]		4.5±2.1	0				Refuted
1994	Wolraich		[*72*] *, * [*73*] *, * [*74*]	5,8±0	5	[*71*]	27.8	16	Confirmed
1996	Lahoste	[*31*] *, * [*32*] *, * [*33*] *, * [*34*] *, * [*35*] *, * [*36*] *, * [*37*] *, * [*38*] *, * [*39*] *, * [*40*] *, * [*41*] *, * [*42*] *, * [*43*] *, * [*44*] *, * [*45*]		4.8±4.3	0	[*46*] *, * [*47*]	3.9, 3.8	0	Attenuated
1996	Safer	[*75*] *, * [*76*]	[*77*] *, * [*78*] *, * [*80*] *, * [*79*]	13.2±19	9				Mostly confirmed
1998	Vaidya	*No ref*	*No ref*		0				Confirmation unlikely
1999	Biederman	[*82*] *, * [*83*] *, * [*84*] *, * [*86*] *, * [*14*] *, * [*13*]	[*85*]	5.5±3.0	11	[*81*]	5.8	10	Attenuated
1999	Dougherty	[*48*] *, * [*49*] *, * [*50*] *, * [*51*] *, * [*52*] *, * [*53*] *, * [*54*] *, * [*55*] *, * [*56*] *, * [*57*] *, * [*58*]		8.1±7.4	3	[*59*]	11.4	0	Refuted
1999	Gainetdinov	[*60*] *, * [*61*] *, * [*62*]		4.6±1.5	0	[*63*] *, * [*64*]	9.3, 5.5	0	Attenuated
1999	MTA group	[*89*] *, * [*90*] *, * [*91*]	[*88*]	4.2±1.8	3	[*87*]	7.6	0	Attenuated

IF: journal impact factor; echoed.

# : number of newspaper articles echoing the corresponding studies; *Ref: reference number in italic*.

In 1998 Vaidya et al. pointed out a selective effect of methylphenidate (MPH) in ADHD. Using functional magnetic resonance imaging they reported that, during an inhibition task, MPH increased striatal activation in 10 ADHD children but reduced it in six control children [19]. However, the reducing effect of MPH in healthy children has neither been confirmed nor refuted subsequently, according to our systematic search corroborated by Vaidya (personal communication, July 2011). A detailed analysis of the data (see Text S1) shows that they are less conclusive than claimed by the authors in their main conclusion.

**Figure 3:**
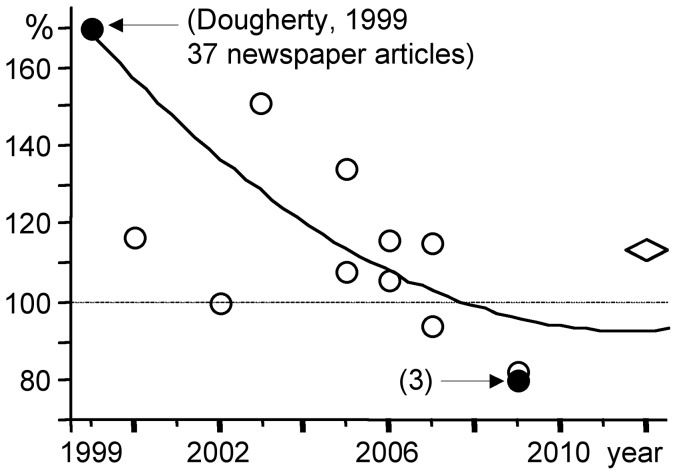
Association between ADHD and the dopamine transporter level in initial and subsequent studies. Dougherty and coworkers (1999) used brain imaging to show that the dopamine transporter was more abundant (+70%) in the striatum of ADHD adults than in control subjects. Subsequent studies did not confirm this observation (data from a meta-analysis [59]). The effect calculated by this meta-analysis is indicated by a diamond. Only one subsequent study (filled circle) was echoed in newspapers (number of articles in parentheses). Empty symbols indicated that the corresponding scientific article has not been echoed by newspapers.

In 1999 Fishman's group used PET to measure the density of the dopamine transporter (DAT) in 6 ADHD adults and 30 healthy controls [20]. They reported that DAT density was elevated by 70% in the striatum of ADHD adults. However, 11 subsequent studies [48]–[58] and a meta-analysis [59] support the conclusion that the DAT density in the striatum and/or its subdivisions is not obviously altered in ADHD patients (Fig. 3 and Table 2).

Gainetdinov et al. proposed for the first time that genetically modified mice lacking DAT might represent a model of ADHD because they exhibited increased locomotion that was decreased in response to psychostimulants [25]. They reported that this “calming” effect was not related to dopamine and might involve the serotonin (5-HT) transporter because the same effect was observed with the antidepressant drug fluoxetine, a specific inhibitor of 5-HT uptake. However, two previous studies [60], [61] and a subsequent one [62] reported that antidepressants that specifically inhibit the 5-HT transporter do not improve ADHD symptoms. Two recent meta-analyses about ADHD medication in children [63] and adults [64] do no recommend drugs targeting 5-HT neurotransmission.

### Scientific follow-up of other “top 10” publications

Four “top 10” publications reported on behavioral observations in humans and two articles corresponded to initial studies. In an initial 1993 study conducted in adults and children, Hauser et al. reported that “subjects with generalized resistance to thyroid hormone (RTH, a genetic disease) have a markedly increased frequency of ADHD as compared to their unaffected family members” [21]. However, three subsequent studies failed to find evidence of RTH in large samples of children and adolescents with ADHD [65]–[67]. Moreover, the association between untreated RTH and ADHD has been further questioned (Table 2 and Text S1) [68]–[70].

In 1994 Wolraich et al. reported that diets high in sucrose or aspartame did not affect the behavior and cognitive performance of 25 pre-school children and 23 school-age children described by their parents as sensitive to sugar [22]. According to a meta-analysis [71], these observations were consistent with previous studies, including three independent reports published between 1990 and 1994 [72]–[74]. This question has not been further investigated (Table 2 and Text S1).

According to the US Drug Enforcement Administration the production of MPH in the US increased nearly six-fold from 1990 to 1995 [26]. Whether or not this huge increase accurately reflected the expansion of MPH treatment was a matter of debate in the 1990s. According to Safer et al. (1996) there occurred a 2.5-fold increase in the prevalence of MPH treatment of youths with ADHD from 1990 to 1995 [26]. This estimate was less alarming than a previous one [75]. Moreover, Safer's data have been questioned by a subsequent study [76]. However, Safer and colleagues used another approach to confirm their original estimate [77] and three studies by two independent groups also reported estimates consistent with Safer's study [78]–[80] (Table 2).

**Figure 4:**
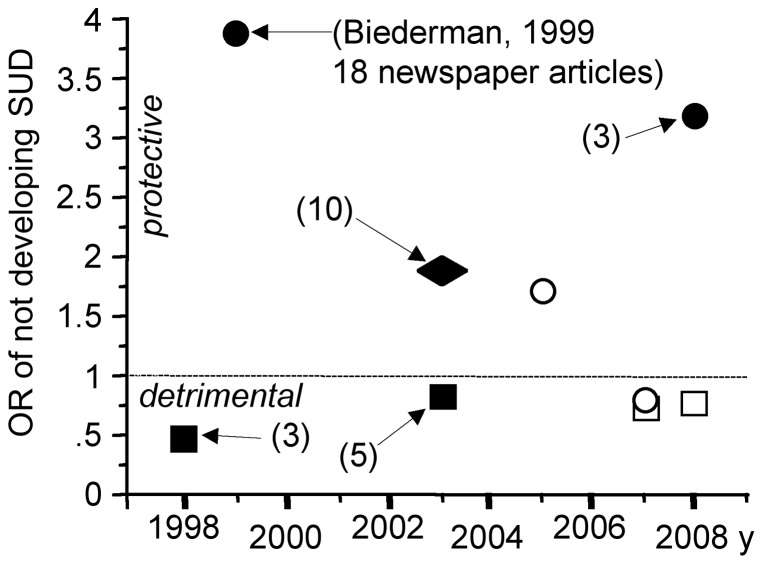
Impact of ADHD pharmacotherapy on later substance use disorder (SUD). According to the “top 10” study published by Biederman et al. in 1999, ADHD children treated with psychostimulants are at a 4-fold reduced risk of later SUD at adolescence. This study and two studies published in 1998 and 2003 were taken into account in a meta-analysis (corresponding OR indicated by a diamond). Circles indicate pharmacotherapy outcome for SUD at adolescence and squares at adulthood. Scientific studies echoed by newspapers are indicated by filled symbols (number of articles in parentheses). Empty symbols indicate that the corresponding scientific article has not been echoed by newspapers.

In 1999 Biederman et al. published a study showing that pharmacotherapy of ADHD reduces the risk for later development of substance use disorder (SUD) [23]. In 2003 the same group published a meta-analysis supporting the same conclusion although with a smaller effect size [81]. This meta-analysis included several studies that were not published in peer-reviewed journals and three studies reporting either an enhanced SUD risk [82], a protective effect [23] or no effect [83]. Subsequent studies either reported a protective effect [84], [85] or no effect [13], [14], [86]. Among all available studies, the “top 10” publication reported the largest protective effect of pharmacotherapy (Fig. 4). However, the same group concluded in 2008: “the findings revealed no evidence that stimulant treatment increases or decreases the risk for subsequent SUD in children and adolescents with ADHD when they reach young adulthood” [13].

Given public concerns regarding psychostimulants and lack of evidence to guide long-term treatment of ADHD, the National Institute of Mental Health sponsored in 1992 a randomized clinical trial, the Multimodal Treatment Study of Children with ADHD (MTA) [24]. A group of 579 children with ADHD were randomly assigned for 14 months to medication management, intensive behavioral therapy, the two combined treatments, or standard community care. “For most ADHD symptoms children in the medication management and combined treatment showed greater improvement than those given behavioral treatment and community care” [24]. Because MTA is the only study in which large groups of children were randomly assigned to different treatments [87], it should be considered as an initial study. Its main conclusion has been confirmed by a smaller study [88]. However, the children enrolled in the MTA study were reassessed 10 [89] and 22 [90] months after the end of the initial study. This follow-up showed a progressive and complete loss of superiority of the medication strategy despite maintenance of psychostimulant treatment [91].

### Summary of the scientific follow-up of each “top 10” publication

References of all studies related to each “top 10” publication are given in Table 2. Among these “top 10” publications, three were refuted either by the same group [17], [21] or by other researchers [20]. The main conclusion in four was strongly attenuated by subsequent studies [18], [23]–[25]. One was neither confirmed nor refuted, but its main conclusion appears very unlikely [19]. The conclusion of one publication [26] has been questioned by one previous and one subsequent publication and confirmed by four others. Finally, one publication has been fully confirmed by a meta-analysis and not questioned subsequently [22].

### Media articles reporting on meta-analyses related to “top 10” publications

Relevant meta-analyses were found for six “top 10” publications, but only two were echoed by newspapers. The 1994 study by Wolraich et al. showing that sugar does not significantly affect child behavior has been confirmed by a meta-analysis published by the same group. This subsequent meta-analysis was also echoed by 16 newspaper articles that repeated Wolraich's conclusion (Table 2). The 1999 study by Biederman et al. was included in a meta-analysis published in 2003 by the same investigators [81]. This meta-analysis was echoed by 10 newspapers articles stating that psychostimulant treatment does not lead to drug abuse. Moreover, seven out of these 10 articles added that medication exerts a protective effect: “ADHD medication resulted in an almost two-fold reduction in the risk of future substance abuse” (*The Sydney Morning Herald*). None, however, mentioned that the size of this protective effect (1.8) was smaller than that reported in the corresponding “top 10” study (3.9) (Fig. 4).

### Media articles reporting on scientific publications related to “top 10” publications

Few scientific publications related to “top 10” publications were echoed by newspapers (Table 2). Indeed, the subsequent scientific studies related to five “top 10” publications [17]–[25] received no media coverage at all. Among the articles subsequent to the 1999 study by Dougherty et al. only one study [56] was echoed by three newspaper articles (Fig. 3). These newspaper articles stated that DAT density was lower than normal in ADHD patients but did not mention that this subsequent study refuted the initial claim. The 1996 epidemiologic study published by Safer et al. [26] was questioned by LeFever et al. [76] and this subsequent study was echoed by 9 newspaper articles. All nine discussed whether ADHD was overdiagnosed, but did not discuss the initial data, i.e. the amplitude of the increase in the prevalence of MPH treatment between 1990 and 1995 [26]. The 1994 study by Wolraich et al. [22] investigating the effect of sugar on child behavior was not followed by subsequent research, but it was preceded by three studies in the early 1990s. Two of these three studies [72], [74] were echoed by four and one newspapers, respectively. Because all four scientific studies concluded that sugar ingestion does not significantly affect child behavior, all newspaper articles reporting on them put forward the same conclusion.

Few scientific publications related to “top 10” publications were echoed by newspapers (Table 2). Indeed, the subsequent scientific studies related to five “top 10” publications [17]–[25] received no media coverage at all. Among the articles subsequent to the 1999 study by Dougherty et al. only one study [56] was echoed by three newspaper articles (Fig. 3). These newspaper articles stated that DAT density was lower than normal in ADHD patients but did not mention that this subsequent study refuted the initial claim. The 1996 epidemiologic study published by Safer et al. [26] was questioned by LeFever et al. [76] and this subsequent study was echoed by 9 newspaper articles. All nine discussed whether ADHD was overdiagnosed, but did not discuss the initial data, i.e. the amplitude of the increase in the prevalence of MPH treatment between 1990 and 1995 [26]. The 1994 study by Wolraich et al. [22] investigating the effect of sugar on child behavior was not followed by subsequent research, but it was preceded by three studies in the early 1990s. Two of these three studies [72], [74] were echoed by four and one newspapers, respectively. Because all four scientific studies concluded that sugar ingestion does not significantly affect child behavior, all newspaper articles reporting on them put forward the same conclusion.

The conclusion of the 1999 MTA study [24] was attenuated by three subsequent studies, of which two [89], [90] were echoed by two and one newspaper articles, respectively. This latter article was the only newspaper article mentioning that a “top 10” finding has been attenuated by the corresponding subsequent study. Indeed, in the *Washington Post* (July 31, 2007) the journalist wrote: “The study [by Jensen and coworkers] is a follow-up to a landmark NIMH study published in 1999. In the earlier phase of the study, nearly 600 children ages 7 to 9 with ADHD were randomly assigned to one of four treatments for 14 months. Those whose medication was managed by an ADHD specialist and those whose treatment involved both drugs and behavioral therapy did far better than those treated by a family physician or with behavior therapy alone. But at the three-year mark, kids from all four groups showed the same amount of improvement.”

The only “top 10” publication that gave rise to a significant public controversy is that by Biederman et al. [23]. Among the seven scientific publications related to this 1999 study about the effect of MPH treatment of ADHD children on later SUD risk, three were echoed by newspapers (Fig. 4). When the study by Lambert and Hartsough [82] was published in 1998, three newspaper articles soon echoed its main conclusion: “Children on Ritalin are three times more likely to develop a taste for cocaine” (*New York Post*, December 8, 1998). In 1999, 13 newspaper articles again cited Lambert's study when they echoed Biederman's study. Six of them supported the view that Biederman's study refuted Lambert's study whereas the seven others gave a neutral report of both studies. Regarding the six scientific studies subsequent to 1999 Biederman' study, two [83], [85] were echoed by five and three newspaper articles, respectively. All these eight press articles put forward this type of statement: “Children who take stimulants to treat ADHD are at no greater risk for using illegal drugs when they are teenagers or adults than children who are not treated with such drugs” (*New York Daily News*, January 6, 2003). Moreover, the three press articles reporting on the study by Wilens et al. (2008) added that stimulant treatment may have a protective effect.

### Media coverage and journal impact factor

**Figure 5:**
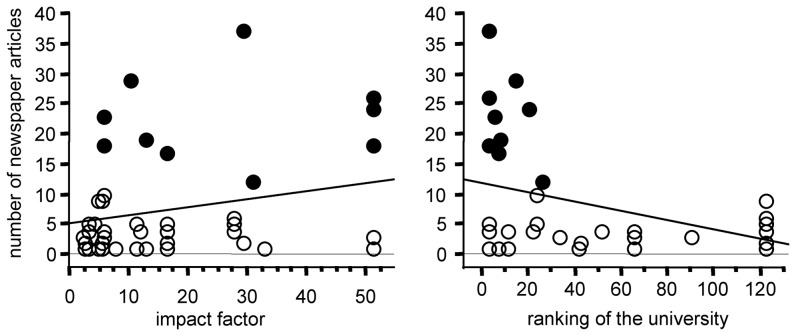
Newspaper coverage of the 47 scientific studies related to ADHD between 1990 and 1999. The number of newspaper articles reporting on each scientific study is expressed as a function of the impact factor of the scientific journal publishing the scientific study (left), or the ranking of the university in which the study was performed (right). The ranking for medicine and pharmacy was that given by ARWU in 2010 (“Shanghai Ranking”). The relationship with the impact factor is not statistically significant (R^2^ = 0.06 ANOVA: f = 3.0 p = 0.09) whereas that with the university ranking is significant (R^2^ = 0.20 ANOVA: f = 11.2 p = 0.0017). The “top10” articles are indicated by filled circles.

In our initial search of scientific publications related to ADHD published in the 1990s and echoed by newspapers we found 47 studies. On average, the impact factor of the journals that published the “top 10” studies (26.6±6.0 mean ± SEM) is larger than for the 37 remaining publications (15.0±2.3 mean ± SEM) and this difference is statistically significant (unpaired *t* test: p = 0.038). However, we observe no significant relationship between the impact factor of the corresponding journal and the number of newspaper articles reporting on these 47 publications (Fig. 5). When we pool the 47 initial publications with their 67 related publications we obtain two doubloons and 112 distinct publications. When we compare the average impact factor of the 56 scientific publications that were echoed by newspapers (17.1±2.1 mean ± SEM) to that of the 56 publications that were not echoed (6.4±1.0 mean ± SEM), the difference is highly significant (unpaired *t* test: p<0.0001).

**Figure 6:**
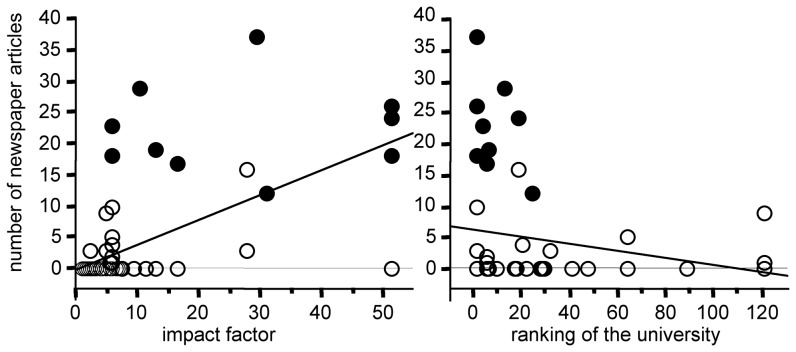
Newspaper coverage of “top 10” publications and of their 67 related scientific studies. The number of newspaper articles reporting on each scientific study is expressed as a function of either the impact factor of the scientific journal publishing the scientific study (left), or the ranking of the university in which the study was performed (right). The relationship with the impact factor (R^2^ = 0.33 ANOVA: f = 36.8 p<0.0001) and with the university ranking (R^2^ = 0.10 ANOVA: f = 8.4 p = 0.005) are statistically significant. The “top10” publications are indicated by filled circles.

When we compare the media coverage of our “top 10” publications to that of the 67 related scientific articles, including meta-analyses (Tables 1 and 2), the difference is huge and seems related to the fact that the averaged impact factor of the related publications is almost always lower than that of the corresponding “top 10” article. More precisely, we observe a strong positive relationship between the magnitude of the newspaper coverage and the impact factor of the corresponding scientific publication (Fig. 6). There are two notable exceptions however. The increase in the prevalence of MPH treatment reported in 1996 by Safer et al. [26] was less alarming than that published in 1995 by Swanson et al. [75]. This previous study was published in a high impact factor journal (51.4), but was not echoed by newspapers, whereas Safer's study received wide media coverage although it appeared in a journal with a much lower impact factor (5.8). Likewise, Biederman et al. reported in 1999 a large protective effect of pharmacotherapy on later SUD risk [23], but reported a null effect in 2008 [13]. Although the latter publication appeared in a journal with a higher impact factor (11.4 compared to 5.8), only the former was echoed by newspapers.

### Media coverage and university ranking

When considering the 47 scientific publications of our initial search that newspapers echoed during the 1990s, we observe a positive relationship between the prestige of the university in which the study was performed and the number of newspaper articles reporting on it (Fig. 5). This relationship is much more significant (p = 0.0017) than that with the journal impact factor. Regarding the “top 10” publications and their 67 related studies, we also observe a positive relationship between the prestige of the university in which the study was performed and the number of newspaper articles reporting on it (Fig. 6).

## Discussion

### Comments

Among “top 10” publications only two studies passed the test of the years, three studies were fully refuted, four were substantially attenuated by subsequent articles and one was not confirmed or refuted though its main conclusion appears unlikely. These seemingly astonishing proportions are nonetheless quite consistent with the devaluation trend of biomedical findings described by Ioannidis and coworkers [6]–[8]. Previous studies suggest that this devaluation trend stems from the fact that initial observations showing a positive effect are much more often published than those reporting no effect [6], [7], [9]. As a consequence, initial observations are often refuted or attenuated by subsequent studies [6]–[8]. Our findings support this interpretation. Indeed, among the seven “top 10” publications that were refuted or attenuated by subsequent studies, six reported initial observations [17], [18], [20], [21], [24], [25]. In contrast, both “top 10” publications that were subsequently confirmed did not report initial observations [22], [26].

As a whole, “top 10” publications received a much larger press coverage (223 newspaper articles) than the 67 related scientific studies, including meta-analyses (57 newspaper articles). More precisely, scientific studies related to eight “top 10” articles received only marginal media coverage whereas the 12 studies related to Wolraich et al. (1994) and to Biederman et al. (1999) received a total media coverage similar in size to both of their corresponding “top 10” publications. Therefore, in the case of ADHD, our observations are consistent with our prediction based on Ioannidis' studies: subsequent scientific articles that refute or attenuate initial studies are much less echoed by newspapers than initial studies. Moreover, only one of these few newspaper articles mentioned that the subsequent study it echoed actually attenuated the corresponding “top 10” claim. In other words, at least in the case of ADHD, we observed an almost complete amnesia in the newspaper coverage of biomedical findings.

We hypothesized that this much lower coverage of subsequent studies was related to the lower impact factor of the journals that published them. Our observations are strongly consistent with this prediction when we compared the coverage of “top 10” publications with that of their 67 related studies. However, regarding the newspaper coverage of the 47 scientific publications of our initial search, its amplitude is not significantly correlated with the impact factor, but rather with the ranking of the university where the study was performed. This suggests that the publication of a scientific study in a high impact factor journal is a prerequisite, but does not guarantee a strong media coverage. The prestige of the university seems to exert an additional influence. Indeed, famous universities have powerful press offices that may help their researchers obtain press coverage [92].

Scientific knowledge always matures from initial and uncertain findings to validated findings. This process often results from the debate of conflicting opinions in the scientific literature. Accordingly, apart from Wolraich's study, our “top 10” publications were involved in scientific debates. However, their press coverage never reflected these debates, apart from a notable, but restricted, exception: the conflicting observations reported by Lambert and Hartsough (1998) and by Biederman et al. (1999). Therefore, as a whole these newspaper articles put forward scientific findings to defend the view that ADHD is a neurological disease mainly caused by genetic factors and that psychostimulant treatments are safe and effective. This is not to say that all press articles published in the 1990s about ADHD echoed the same line. We found several press articles defending the view that ADHD is mainly a social construct, or a disorder caused by environmental factors, and questioning the safety and effectiveness of psychostimulant medication. However, these press articles usually cited medical opinions but, apart from comments on Lambert and Hartsough's study, were not based on scientific observations published in peer-reviewed journals.

The fact that almost all newspaper articles included in the present study promote a medicalized view of ADHD results largely from our process of selecting articles reporting on biomedical findings. However, in the course of the present study we observed three notable exceptions suggesting a reporting bias favoring a medical conception of ADHD and its treatment. Both “top 10” publications in *Pediatrics* [23], [26] offered conclusions that were attenuated by two related studies, but these studies received no media coverage although they were published in more prestigious journals. The third exception is an example of refutation: six newspaper articles reporting in 1999 on Biederman's study about the protective effect of pharmacotherapy towards SUD [23] pointed out that this “top 10” study refuted a previous publication on the same topic [82]. Although these reporting exceptions may reflect a biased conception of ADHD, our observations cannot tell whether scientists, journalists, or both, were responsible for it.

### Limitations

Because we only focused on ADHD, generalization of our observations to other biomedical domains remains hypothetical. However, the hypothesis that grounded our study originated from the seminal studies by Ioannidis and coworkers [6]–[8]. Because these studies covered a wide range of biomedical topics, our main finding might also hold true regarding most medical conditions: media reporting strongly favors initial studies although most of them are refuted or attenuated by subsequent studies.

We investigated scientific studies and their newspaper coverage published during the 1990s. The 12-year delay between this selected decade and the present study was required to test whether subsequent studies consistently confirmed “top 10” studies. We do not know whether journalists pay more attention to replication of initial findings today than during the 1990s.

We focused on the ten publications related to ADHD that received the widest press coverage from 1990 to 1999. We did not investigate the scientific follow-up of all 47 publications that were echoed at least once. Although this selection might affect our conclusions, it is justified by the fact that the media coverage of the “top 10” publications (223 articles) was nearly seven times larger than that of the 37 others (124 articles).

To quantify media coverage, we considered only newspaper articles published from one day preceding to nine days following the publication of each scientific study. Indeed, when newspaper articles appeared much later than the scientific publication, it was often impossible to identify it with certainty. Consequently, we did not examine the long-term impact of scientific publications in the lay press. However, in relative terms, it is likely that the scientific publications with the strongest long-term impact also received the widest newspaper coverage shortly following their publication.

Here we only investigated the coverage of scientific findings by newspapers. Although generalization of our observations to other media, especially television, is still hypothetical, it is unlikely that television provides more contextualized and in-depth reports than newspapers. Indeed, the televised reporting of discoveries related to genetic diseases appears less accurate than in newspapers [93].

## Conclusions

During the 1990s, press coverage of scientific studies about the biology and etiology of ADHD contributed “to much wider acceptance of the disorder as having neurological and genetic, rather than environmental origins” [16]. Newspaper articles reporting on our “top 10” publications repeatedly claimed that these findings might soon result in improved pharmacological treatments and in commercially available biomarkers to confirm the ADHD diagnosis. None of these promises have yet been fulfilled. Moreover, general agreement now exists among scientists that environmental risk factors play a central role in ADHD etiology [94]–[96]. Because newspapers failed to inform the lay public that most initial scientific claims were later refuted or strongly attenuated, they did not reflect the evolution of scientific knowledge. In turn, because scientific findings echoed by newspapers are more often cited in the scientific literature [97], this biased media coverage probably favors the visibility of initial findings. Therefore, not only the lay public but also a substantial proportion of interested professionals, scientists and clinicians might be influenced by this inaccurate media coverage. This might have detrimental consequences on the management and prevention of ADHD [98].

We showed here, using the example of ADHD, that press coverage of health issues, by strongly favoring initial studies, ignores the publication bias resulting from the devaluation trends of initial findings. If further investigations of other health issues confirm our observations and reinforce our interpretations, it might be timely for scientists, journal editors and university media writers to define and respect ethical rules regarding health science communication. For example, press releases reporting on an initial study should include a warning statement pointing out that these findings must be confirmed by subsequent independent investigations. Indeed, the quality of press releases positively influences the quality of associated newspaper stories [99]. The time would be also right to warn journalists about this major publication bias inherent to the scientific process.

## Supporting Information

Table S1 List of the 47 scientific publications reporting on primary data about ADHD and echoed at least once by newspapers.
(PDF)

Table S2 PubMed search of meta-analyses related to the “top 10” scientific articles.
(PDF)

Text S1 Detailed report on the scientific follow-up of each “top 10” article.
(PDF)
