# Supplementary material for: Why Most Biomedical Findings Echoed by Newspapers Turn Out to be False: The Case of Attention Deficit Hyperactivity Disorder
Source: PLoS One. 2012 Sep 12;7(9):e44275. doi: 10.1371/journal.pone.0044275 (PMC3440402; doi:10.1371/journal.pone.0044275)
Supplement: Table S1 — List of the 47 scientific publications reporting on primary data about ADHD and echoed at least once by newspapers. (PDF) [file pone.0044275.s001.pdf]

Table S1. List of the 47 scientific publications reporting on primary data about ADHD and echoed at least once by newspapers. The first column (#) indicates the number of newspaper articles echoing on each scientific publication.

#	Reference
37	Dougherty DD, Bonab AA, Spencer TJ, Rauch SL, Madras BK, Fischman AJ (1999) Dopamine transporter density in patients with attention deficit hyperactivity disorder. Lancet 354:2132-33
29	Vaidya CJ, Austin G, Kirkorian G, Ridlehuber HW, Desmond JE, Glover GH, Gabrieli JD (1998) Selective effects of methylphenidate in attention deficit hyperactivity disorder: a functional magnetic resonance study. Proc Natl Acad Sci U S A 95:14494-9
26	Hauser P, Zametkin AJ, Martinez P, Vitiello B, Matochik JA, Mixson AJ, Weintraub BD (1993) Attention deficit-hyperactivity disorder in people with generalized resistance to thyroid hormone. N Engl J Med 328:997-1001.
24	Wolraich ML, Lindgren SD, Stumbo PJ, Stegink LD, Appelbaum MI, Kiritsy MC (1994) Effects of diets high in sucrose or aspartame on the behavior and cognitive performance of children. N Engl J Med 330:301-307
23	Safer DJ, Zito JM, Fine EM (1996) Increased methylphenidate usage for attention deficit disorder in the 1990s. Pediatrics 98:1084-1088
19	LaHoste GJ, Swanson JM, Wigal SB, Glabe C, Wigal T, King N, Kennedy JL (1996) Dopamine D4 receptor gene polymorphism is associated with attention deficit hyperactivity disorder. Mol Psychiatry 1:121-124
18	Biederman J, Wilens T, Mick E, Spencer T, Faraone SV (1999) Pharmacotherapy of attention-deficit/hyperactivity disorder reduces risk for substance use disorder. Pediatrics 104:e20.
18	Zametkin AJ, Nordahl TE, Gross M, King AC, Semple WE, Rumsey J, Hamburger S, Cohen RM (1990) Cerebral glucose metabolism in adults with hyperactivity of childhood onset. N Engl J Med 323:1361-1366.
17	The MTA Cooperative Group (1999) A 14- month randomized clinical trial of treatment strategies for attention-deficit/hyperactivity disorder. Arch Gen Psychiatry 56:1073-1086.
12	Gainetdinov RR, Wetsel WC, Jones SR, Levin ED, Jaber M, Caron MG (1999) Role of serotonin in the paradoxical calming effect of psychostimulants on hyperactivity. Science 283:397-401
10	Shaywitz BA, Sullivan CM, Anderson GM, Gillespie SM, Sullivan B, Shaywitz SE (1994) Aspartame, behavior, and cognitive function in children with attention deficit disorder. Pediatrics 93:70-75
9	LeFever GB, Dawson KV, Morrow AL (1999) The extent of drug therapy for attention deficit-hyperactivity disorder among children in public schools. Am J Public Health 89:1359-1364.
9	Nagin D, Tremblay RE (1999) Trajectories of boys' physical aggression, opposition, and hyperactivity on the path to physically violent and nonviolent juvenile delinquency. Child Dev 70:1181-1196
6	Comings DE, Comings BG, Muhleman D, Dietz G, Shahbahrami B, Tast D, Knell E, Kocsis P, Baumgarten R, Kovacs BW, et al. (1991) The dopamine D2 receptor locus as a modifying gene in neuropsychiatric disorders. JAMA 266:1793-1800
5	Castellanos FX, Giedd JN, Marsh WL, Hamburger SD, Vaituzis AC, Dickstein DP, Sarfatti SE, Vauss YC, Snell JW, Lange N, Kaysen D, Krain AL, Ritchie GF, Rajapakse JC, Rapoport JL (1996) Quantitative brain magnetic resonance imaging in attention-deficit hyperactivity disorder. Arch Gen Psychiatry 53:607-616.
5	Hazell PL, McDowell MJ, Walton JM (1996) Management of children prescribed psychostimulant medication for attention deficit hyperactivity disorder in the Hunter region of NSW. Med J Aust 165:477-480
5	Jones TW, Borg WP, Boulware SD, McCarthy G, Sherwin RS, Tamborlane WV (1995) Enhanced adrenomedullary response and increased susceptibility to neuroglycopenia: mechanisms underlying the adverse effects of sugar ingestion in healthy children. J Pediatr 126:171-177
5	Pincus HA, Tanielian TL, Marcus SC, Olfson M, Zarin DA, Thompson J, Magno Zito J (1998) Prescribing trends in psychotropic medications: primary care, psychiatry, and other medical

	specialties. JAMA 279:526-531
5	Weissman MM, Warner V, Wickramaratne PJ, Kandel DB (1999) Maternal smoking during pregnancy and psychopathology in offspring followed to adulthood. J Am Acad Child Adolesc Psychiatry 38:892-899
5	Volkow ND, Wang GJ, Fowler JS, Gatley SJ, Logan J, Ding YS, Hitzemann R, Pappas N (1998) Dopamine transporter occupancies in the human brain induced by therapeutic doses of oral methylphenidate. Am J Psychiatry 155:1325-1331
4	Bachorowski JA, Newman JP, Nichols SL, Gans DA, Harper AE, Taylor SL (1990) Sucrose and delinquency: behavioral assessment. Pediatrics 86:244-253
4	Barkley RA, Murphy KR, Kwasnik D (1996) Motor vehicle driving competencies and risks in teens and young adults with attention deficit hyperactivity disorder. Pediatrics 98:1089-1095
4	Cook EH, Jr., Stein MA, Krasowski MD, Cox NJ, Olkon DM, Kieffer JE, Leventhal BL (1995) Association of attention- deficit disorder and the dopamine transporter gene. Am J Hum Genet 56:993-998.
4	Gillberg C, Melander H, von Knorring AL, Janols LO, Thernlund G, Hagglof B, Eidevall- Wallin L, Gustafsson P, Kopp S (1997) Long-term stimulant treatment of children with attention-deficit hyperactivity disorder symptoms. A randomized, double-blind, placebo-controlled trial. Arch Gen Psychiatry 54:857-864.
4	McCormick MC, Brooks-Gunn J, Workman-Daniels K, Turner J, Peckham GJ (1992) The health and developmental status of very low-birth-weight children at school age. JAMA 267:2204-2208.
4	Musser CJ, Ahmann PA, Theye FW, Mundt P, Broste SK, Mueller-Rizner N (1998) Stimulant use and the potential for abuse in Wisconsin as reported by school administrators and longitudinally followed children. J Dev Behav Pediatr 19:187-192.
4	Tremblay RE, Pihl RO, Vitaro F, Dobkin PL (1994) Predicting early onset of male antisocial behavior from preschool behavior. Arch Gen Psychiatry 51:732-739
4	Wilens TE, Biederman J, Prince J, Spencer TJ, Faraone SV, Warburton R, Schleifer D, Harding M, Linehan C, Geller D (1996) Six- week, double-blind, placebo-controlled study of desipramine for adult attention deficit hyperactivity disorder. Am J Psychiatry 153:1147-1153.
4	Needleman HL, Riess JA, Tobin MJ, Biesecker GE, Greenhouse JB (1996) Bone lead levels and delinquent behavior. JAMA 275:363-369
3	Hack M, Breslau N, Weissman B, Aram D, Klein N, Borawski E (1991) Effect of very low birth weight and subnormal head size on cognitive abilities at school age. N Engl J Med 325:231-237
3	Lambert NM, Hartsough CS (1998) Prospective study of tobacco smoking and substance dependencies among samples of ADHD and non- ADHD participants. J Learn Disabil 31:533-544.
3	Pelham WE, Aronoff HR, Midlam JK, Shapiro CJ, Gnagy EM, Chronis AM, Onyango AN, Forehand G, Nguyen A, Waxmonsky J (1999) A comparison of ritalin and adderall: efficacy and time-course in children with attention-deficit/hyperactivity disorder. Pediatrics 103:e43.
2	Egger J, Stolla A, McEwen LM (1992) Controlled trial of hyposensitisation in children with food-induced hyperkinetic syndrome. Lancet 339:1150-1153.
2	Landgren M, Kjellman B, Gillberg C (1998) Attention deficit disorder with developmental coordination disorders. Arch Dis Child 79:207-212
2	Levy F, Hay DA, McStephen M, Wood C, Waldman I (1997) Attention-deficit hyperactivity disorder: a category or a continuum? Genetic analysis of a large-scale twin study. J Am Acad Child Adolesc Psychiatry 36:737-744.
2	Silberstein RB, Farrow M, Levy F, Pipingas A, Hay DA, Jarman FC (1998) Functional brain electrical activity mapping in boys with attention-deficit/hyperactivity disorder. Arch Gen Psychiatry 55:1105-1112.
1	Needleman HL, Schell A, Bellinger D, Leviton A, Allred EN (1990) The long-term effects of exposure to low doses of lead in childhood. An 11-year follow-up report. N Engl J Med 322:83-88.
1	Boris M, Mandel FS (1994) Foods and additives are common causes of the attention deficit hyperactive disorder in children. Ann Allergy 72:462-468
1	Gill M, Daly G, Heron S, Hawi Z, Fitzgerald M (1997) Confirmation of association between

	attention deficit hyperactivity disorder and a dopamine transporter polymorphism. Mol Psychiatry 2:311-313
1	Hack M, Taylor HG, Klein N, Eiben R, Schatschneider C, Mercuri-Minich N (1994) School-age outcomes in children with birth weights under 750 g. N Engl J Med 331:753-759.
1	Mannuzza S, Klein RG, Bessler A, Malloy P, LaPadula M (1993) Adult outcome of hyperactive boys. Educational achievement, occupational rank, and psychiatric status. Arch Gen Psychiatry 50:565-576
1	Milberger S, Biederman J, Faraone SV, Chen L, Jones J (1996) Is maternal smoking during pregnancy a risk factor for attention deficit hyperactivity disorder in children? Am J Psychiatry 153:1138-1142
1	Pardo JV, Fox PT, Raichle ME (1991) Localization of a human system for sustained attention by positron emission tomography. Nature 349:61-64
1	Rappley MD, Mullan PB, Alvarez FJ, Eneli IU, Wang J, Gardiner JC (1999) Diagnosis of attention-deficit/hyperactivity disorder and use of psychotropic medication in very young children. Arch Pediatr Adolesc Med 153:1039-1045.
1	Stevens LJ, Zentall SS, Deck JL, Abate ML, Watkins BA, Lipp SR, Burgess JR (1995) Essential fatty acid metabolism in boys with attention-deficit hyperactivity disorder. Am J Clin Nutr 62:761-768.
1	Szatmari P, Saigal S, Rosenbaum P, Campbell D, King S (1990) Psychiatric disorders at five years among children with birthweights less than 1000g: a regional perspective. Dev Med Child Neurol 32:954-962
1	Weitzman M, Gortmaker S, Sobol A (1992) Maternal smoking and behavior problems of children. Pediatrics 90:342-349
