# Supplementary material for: Why Most Biomedical Findings Echoed by Newspapers Turn Out to be False: The Case of Attention Deficit Hyperactivity Disorder
Source: PLoS One. 2012 Sep 12;7(9):e44275. doi: 10.1371/journal.pone.0044275 (PMC3440402; doi:10.1371/journal.pone.0044275)
Supplement: Table S2 — PubMed search of meta-analyses related to the “top 10” scientific articles. (PDF) [file pone.0044275.s002.pdf]

Table S2. PubMed search of meta-analyses related to the "top 10" scientific articles. The Boolean equation was: (ADHD OR attention deficit) AND (specific term). This PubMed search was limited to "human" and "meta-analysis". When several meta-analyses were found, the two most recent were selected. Reference number is indicated in italic

Year 1st author	Title	Specific term	meta-analysis found
1990 Zametkin	Cerebral glucose metabolism in adults with hyperactivity of childhood onset	cerebral glucose metabolism OR imaging	No meta-analysis found, but claim refuted by the same group.
1993 Hauser	Attention deficit-hyperactivity disorder in people with generalized resistance to thyroid hormone	thyroid	No meta-analysis found, but claim refuted by the same group.
1994 Wolraich	Effects of diets high in sucrose or aspartame on the behavior and cognitive performance of children	sugar	*71*
1996 LaHoste	Dopamine D4 receptor gene polymorphism is associated with attention deficit hyperactivity disorder	D4 OR DRD4	*46, 47*
1996 Safer	Increased methylphenidate usage for attention deficit disorder in the 1990s	methylphenidate AND (prevalence OR usage)	No meta-analysis found
1998 Vaidya	Selective effects of methylphenidate in attention deficit hyperactivity disorder: a fMRI study	fMRI OR MRI OR magnetic resonance	No meta-analysis found
1999 Biederman	Pharmacotherapy of attention-deficit/hyperactivity disorder reduces risk for substance use disorder	substance	*81*
1999 Dougherty	Dopamine transporter density in patients with attention deficit hyperactivity disorder	dopamine AND transporter	*59*
1999 Gainetdinov	Role of serotonin in the paradoxical calming effect of psychostimulants on hyperactivity	medication	*63, 64*
1999 MTA group	A 14-month randomized clinical trial of treatment strategies for ADHD	treatment OR treatments	*87*
