# Supplementary material for: Why Most Biomedical Findings Echoed by Newspapers Turn Out to be False: The Case of Attention Deficit Hyperactivity Disorder
Source: PLoS One. 2012 Sep 12;7(9):e44275. doi: 10.1371/journal.pone.0044275 (PMC3440402; doi:10.1371/journal.pone.0044275)
Supplement: Text S1 — Detailed report on the scientific follow-up of each “top 10” article. (PDF) [file pone.0044275.s003.pdf]

Text S1
This text is a detailed account of the scientific follow-up of each "top 10" article. References indicated by numbers in brackets are those given in the reference list of the article. Additional references are given after the text in alphabetic order.

Subsequent scientific studies of neurobiological observations in humans

All four publications that provided neurobiological data in humans were initial studies [17-20]. Indeed, according to the authors and in agreement with our own searches, the specific questions under investigation were not previously tackled.

In 1990 Zametkin and coworkers used positron-emission tomography (PET) to measure cerebral glucose metabolism in 25 adults with ADHD and in 50 normal adults while they performed an auditory task [17]. They reported that mean global cerebral glucose metabolism was 8.1 % lower in the ADHD adults than in the normal controls [17]. Only four subsequent studies, all performed by Zametkin's group, used the same technique to investigate whether ADHD patients differ from healthy controls. In 1993 they failed to show significant differences between 10 ADHD teenagers and 10 controls [27]. Absence of differences between 15 ADHD boys and 13 control boys was later confirmed, although a significant difference was found regarding girls [28]. This latter difference, however, was not confirmed in a study with a larger sample [29]. Finally, the initial study comparing ADHD adults to healthy controls was disconfirmed by another study based on a larger number of subjects and controlling for the effect of age on cerebral glucose metabolism [30]. Altogether, these four subsequent studies fully disconfirmed the initial finding.

LaHoste and coworkers showed in 1996 that a specific allele (called "7R") of the gene coding for the D4 dopamine receptor was present in 49% of ADHD children and in only 21% of the healthy control children [18]. Subsequent investigations of this case-control association, however, did not confirm this large difference (Fig. 2). Indeed, meta-analyses have repeatedly concluded that the 7R allele confers a statistically significant, but small, risk: it is present in 23% of ADHD children and in 17% of controls [46, 47]. Table 2 lists the 15 subsequent studies included in the most recent meta-analysis.

In 1998 Vaidya et al. pointed out a selective effect of methylphenidate (MPH) in ADHD. Using functional magnetic resonance imaging they reported that, during an inhibition task, MPH increased striatal activation in 10 ADHD children but reduced it in six control children [19]. Regarding ADHD children this enhancing effect of MPH has been confirmed by subsequent studies (Shafritz et al., 2004; Rubia et al., 2011) However, the reducing effect of MPH in healthy children has neither been confirmed nor refuted subsequently, according to our systematic search corroborated by Vaidya (personal communication, July 2011). Moreover, the initial observations are less robust than stated by Vaidya et al. in their summary and conclusion. Indeed, among the 10 ADHD children the enhancing effect of MPH was observed in only 8 children whereas a depressing effect was reported regarding the two others. Moreover, among the 6 control children and 3 unaffected siblings tested, the depressing effect of MPH was only observed in 6 children whereas an enhancing effect was shown in the 3 others [19]. Therefore, it seems very unlikely that these findings might lead to a valid biological test of the ADHD diagnosis.

In 1999 Fishman's group used PET to measure the density of the dopamine transporter (DAT) in 6 ADHD adults and 30 healthy controls [20]. They reported that DAT density was elevated by 70 % in the striatum of ADHD adults. However, 4 of the 6 ADHD subjects had a previous history of stimulant therapy [58]. Fishman's group published two subsequent studies on the same issue. In 2005 they reported that DAT density was enhanced by 34% in the striatum of 6 ADHD adults compared to 6 healthy controls [58]. In their most recent and extensive study the same workers studied 26 healthy adults and 21 ADHD adults who had never been medicated with psychostimulants [57]. They observed no significant differences regarding the DAT density in either putamen or left caudate, but a 15% greater DAT density in the right caudate of ADHD patients compared to controls [57]. The same issue was further investigated by seven independent groups in Germany, Korea, Sweden and USA. These studies reported either no differences between ADHD subjects and controls [52-54], a moderately elevated striatal DAT density [48-51] or a moderately depressed level in both caudate and putamen [55, 56] and in the ventral striatum (Volkow et al. 2010). Altogether, these studies support the conclusion that the DAT density in the striatum and/or its subdivisions is not obviously altered in

ADHD patients (Fig. 3). A meta-analysis including all published observations showed that striatal DAT density in ADHD patients actually depends on previous psychostimulant exposure: higher density is associated with a history of medication [59]. According to recent studies, in drug-naive ADHD patients the striatal DAT density is no different or slightly lower than among normal control subjects [56, 59].

Previous and subsequent scientific studies of behavioral observations in humans

Four of our "top 10" publications reported on behavioral observations in humans and two articles corresponded to initial studies. In an initial 1993 study conducted in adults and children, Hauser et al. reported that "subjects with generalized resistance to thyroid hormone (RTH) have a markedly increased frequency of ADHD as compared to their unaffected family members" [21]. Because RTH is a rare disease of genetic origin, they concluded: "Although it is unlikely that a substantial percentage of ADHD patients also have RTH, prospective studies of thyroid function should be undertaken in these patients." Three subsequent studies, however failed to find evidence of RTH in large samples of children and adolescents with ADHD [65-67]. Another group of investigators showed that low intelligence, but not ADHD, is associated with untreated RTH [68]. In 1997 Hauser et al. further investigated ADHD symptoms in a larger sample of adults and children suffering from RTH. They observed that hyperactivity defined by DSM-IV criteria was significantly more frequent in RTH patients than in unaffected family members whereas inattention was equally infrequent in both groups [69]. Given that attention deficit is by far the most frequent symptom observed in adults with ADHD (Kessler et al., 2010), the initial study erroneously associated ADHD with RTH, at least regarding adults. Finally, Hauser et al. suggested in the initial study: "There could be a role for thyroid hormone in the treatment of ADHD" [21]. A later study showed that treatment with thyroid hormone improves hyperactivity in patients with both ADHD and RTH, but reported that it is either ineffective or detrimental in patients with ADHD only [70]. In sum, RTH is a genetic thyroid disorder that mimics hyperactive symptoms of ADHD, but "current data do not support a genetic linkage of RTH with ADHD (Pearl et al., 2001).

In 1994 Wolraich et al. reported that diets high in sucrose or aspartame did not affect the behavior and cognitive performance of 25 pre-school children and 23 school-age children described by their parents as sensitive to sugar [22]. According to a meta-analysis [71], these observations were consistent with previous studies, including three independent reports published between 1990 and 1994 [72-74]. To our knowledge, and in agreement with a recent article [105], the effect of short-term (i.e. a few weeks) diets high in sucrose or aspartame has not been further investigated. Available data rule out a significant contribution of such diets to ADHD symptoms. However, two naturalistic studies reported that hyperactivity score was positively associated either with prolonged consumption of soft drinks by adolescents [105] or of "junk food" (i.e. high in fat and sugar) by pre-school children [106]. As stated by their authors, these observations do not prove a causal link between high sugar consumption and ADHD. For example, high intakes of soft drink and "junk food" are also associated with higher caffeine consumption, lower fruit and vegetable intakes and parenting style (Lien et al., 2006; Wiles et al., 2009).

In 1999 Biederman et al. published a study showing that pharmacotherapy of ADHD reduces the risk for later development of substance use disorder (SUD) [23]. In 2003 the same group published a meta-analysis supporting the same conclusion although with a smaller effect size [81]. This meta-analysis included several studies that were not published in peer-reviewed journals and three studies reporting either an enhanced SUD risk [82], a protective effect [23] or no effect [83]. Subsequent studies either reported a protective effect of pharmacotherapy [84, 85] or no effect [13, 14, 86]. In their meta-analysis Wilens et al. already noted that the protective effect of pharmacotherapy towards later development of SUD was larger when tested at adolescence than when ADHD children reach adulthood. This view is consistent with recent observations: two studies in adolescents reported a protective effect in boys [84] and girls [85] whereas two studies in adults [13, 86] and one in adolescents [14] reported no effects. Among all available studies, the 1999 publication by Biederman et al. reported the largest protective effect of pharmacotherapy (Fig. 4). However, the same group concluded in 2008: "the findings revealed no evidence that stimulant treatment increases or decreases the risk for subsequent SUD in children and adolescents with ADHD when they reach young adulthood" [13].

Given public concerns regarding psychostimulants and lack of evidence to guide long-term treatment of ADHD, the National Institute of Mental Health sponsored in 1992 a randomized clinical trial, the Multimodal Treatment Study of Children with ADHD (MTA). A group of 579 children with ADHD were randomly assigned for 14 months to medication management, intensive behavioral therapy, the two combined treatments, or standard community care. "For most ADHD symptoms children in the medication management and combined treatment showed greater improvement than those given behavioral treatment and community care" [24]. Combined treatment did not yield significant greater benefits than medication management for core ADHD symptoms, but may have provided modest advantages for non-ADHD symptoms, social skills and academic performances. According to a recent meta-analysis, MTA is the only study in which large groups of children were randomly assigned either to behavioral treatment or to medication and compared for ADHD symptoms [87]. Because of this substantial methodological difference, MTA should be considered as an initial study. In 2008 Pelham and Fabiano reviewed the treatment literature on ADHD [12] and cited only 2 published studies comparing groups of ADHD children treated either with psychostimulants or behavioral therapy [24, 88]. Both studies reached the same conclusion that psychostimulant medication is superior to behavioral therapies regarding ADHD core symptoms, but MTA was by far the most extensive study in terms of treatment duration and number of enrolled children. However, the children enrolled in the MTA study were reassessed 10 [89] and 22 [90] months after the end of the initial study. At 10 months the medication strategy showed a smaller but still significant superiority over behavioral therapy [89]. However, at 22 months, treatment groups did not differ significantly on any measure (ADHD symptoms, social skills, reading scores and non-ADHD symptoms) [90]. This continued loss of superiority occurred despite maintenance of psychostimulant medication during follow-up [91].

Prevalence of methylphenidate usage from 1990 to 1995

According to the US Drug Enforcement Administration the production of MPH in the US increased nearly six-fold from 1990 to 1995 [26]. Whether or not this huge increase accurately reflected the expansion of MPH treatment was a matter of debate in the 1990s. According to Safer et al. (1996) there occurred a 2.5-fold increase in the prevalence of MPH treatment of youths with ADHD from 1990 to 1995 [26]. Their conclusion rested on a count of all public school students receiving ADHD medication in Baltimore County, Maryland, on an analysis of the Maryland Medicaid data and on indirect national databases. However, Swanson et al. (1995) calculated from a national commercial database that the number of patients receiving a MPH prescription grew from 526,000 in 1990 to 1290,000 in 1993 [75]. This 2.45-fold increase is larger than that reported by Safer et al. for the same three years (i.e. 1.78-fold) [26]. In their article Safer et al. cited Swanson's study without noting the quantitative differences. Moreover, Safer et al. also (1996) calculated that approximately 2.8% of US youths aged 5 to 18 years were receiving MPH in 1995. This latter estimate was questioned by LeFever et al. (1999) who observed in two cities of Virginia that 8% to 10% of public school children were receiving ADHD medication in 1995 [76]. LeFever et al. (1999) argued that Safer's study "emphasized data from low-distributions states; therefore, their findings may not reflect ADHD treatment trends across the nation" [76]. Indeed, large geographic variations in the prevalence of stimulant medication were reported in the 1990s (Cox et al., 2003). However, using a national database, Safer and colleagues confirmed their original estimate of the increase in MPH prescription to US youths [77]. Studies by two other independent groups estimating the US national trend in the prevalence of MPH treatment in youths in the mid-1990s also reported estimates consistent with Safer's data [78-80].

Role of serotonin in psychostimulant medication of ADHD

Gainetdinov et al. proposed a mouse model of ADHD and showed that their genetically modified mice exhibited increased locomotion that was decreased in response to psychostimulants [25]. They reported that this “calming” effect might involve the serotonin (5-HT) transporter because the same effect was observed with fluoxetine, a specific inhibitor of 5-HT uptake. They suggested that "hyperkinetic behaviors may be controlled through the precise targeting of 5-HT receptors or even through enhanced availability of 5-HT precursors" [25]. However, Volkow et al. (2000) questioned these interpretations, pointing out that MPH is a weak inhibitor of the 5-HT transporter and that SSRI

antidepressants, which specifically inhibit this transporter, do not improve ADHD symptoms, as reported by previous studies [60, 61]. A recent meta-analysis of ADHD treatment in adults [64] identified only one double-blind placebo-controlled trial comparing one SSRI antidepressant (paroxetine) to a psychostimulant. According to this study, paroxetine is clearly not effective for ADHD [62]. Concerning the pharmacotherapy of ADHD children, the most recent meta-analysis found no placebo-controlled trials of SSRI antidepressants [63].

Cox ER, Motheral BR, Henderson RR, Mager D (2003) Geographic variation in the prevalence of stimulant medication use among children 5 to 14 years old: results from a commercially insured US sample. Pediatrics 111: 237-243.

Kessler RC, Green JG, Adler LA, Barkley RA, Chatterji S et al. (2010) Structure and diagnosis of adult attention-deficit/hyperactivity disorder: analysis of expanded symptom criteria from the Adult ADHD Clinical Diagnostic Scale. Arch Gen Psychiatry 67: 1168-1178.

Lien L, Lien N, Heyerdahl S, Thoresen M, Bjertness E (2006) Consumption of soft drinks and hyperactivity, mental distress, and conduct problems among adolescents in Oslo, Norway. Am J Public Health 96: 1815-1820.

Pearl PL, Weiss RE, Stein MA (2001) Medical mimics. Medical and neurological conditions simulating ADHD. Ann N Y Acad Sci 931: 97-112.

Rubia K, Halari R, Mohammad AM, Taylor E, Brammer M (2011) Methylphenidate normalizes frontocingulate underactivation during error processing in attention-deficit/hyperactivity disorder. Biol Psychiatry 70: 255-262.

Shafritz KM, Marchione KE, Gore JC, Shaywitz SE, Shaywitz BA (2004) The effects of methylphenidate on neural systems of attention in attention deficit hyperactivity disorder. Am J Psychiatry 161: 1990-1997.

Wiles NJ, Northstone K, Emmett P, Lewis G (2009) 'Junk food' diet and childhood behavioural problems: results from the ALSPAC cohort. Eur J Clin Nutr 63: 491-498.

Volkow ND, Gatley SJ, Fowler JS, Wang GJ, Swanson J (2000) Serotonin and the therapeutic effects of ritalin. Science 288: 11.

Volkow ND, Wang GJ, Newcorn JH, Kollins SH, Wigal TL et al. (2010) Motivation deficit in ADHD is associated with dysfunction of the dopamine reward pathway. Mol Psychiatry doi:10.1038/mp.2010.97.
